# Improvement of dielectric performance of solid/gas composite insulation with YSZ/ZTA coatings

**DOI:** 10.1038/s41598-019-40515-8

**Published:** 2019-03-07

**Authors:** Zhu Sun, Weiwei Fan, Zhiyuan Liu, Yu Bai, Yingsan Geng, Jianhua Wang

**Affiliations:** 1 State Key Laboratory of Electrical Insulation and Power Equipment, Xi’an Jiaotong University, Xi’an, 710049 People’s Republic of China; 2 Department of Nuclear Science and Engineering, Massachusetts Institute of Technology, Cambridge, 02139 USA; 3 State Key Laboratory for Mechanical Behavior of Materials, Xi’an Jiaotong University, Xi’an, 710049 People’s Republic of China

## Abstract

N_2_ has been introduced to gas-insulated switchgears (GIS) as an environmentally friendly insulation medium for SF_6_ gas. Considering the low breakdown strength of N_2_, it’s essential to apply a dielectric coating on part of the electrode in GIS. In the current work, yttria-stabilized zirconia (YSZ) and zirconia-toughened alumina (ZTA) ceramics are tactfully applied as dielectric coating materials and successfully coated on the surface of rod electrode via high efficiency supersonic atmospheric plasma spraying method. It is demonstrated that both YSZ and ZTA have excellent thermal stability. In the measured frequency range 10^−2^–10^6^ Hz, the dielectric constant (*ε*′) decreases with the increase in frequency. At measured temperatures (0–140 °C), *ε*′ increases from 15.45 to 16.31 for YSZ ceramic at 1 kHz, while it varies between 30.23 and 39.34 for ZTA ceramic. As compared to that of bare electrode, the 50% BDV (U_50_) of solid/gas composite insulation system is significantly improved when using YSZ and ZTA as coating materials. Furthermore, the U_50_ increases with the increase of coating thickness. For the electrode coated with ZTA ceramic (500 μm), the U_50_ value reaches to 86 kV, which is enhanced about 21.13% in comparison with that of bare electrode. With respect to YSZ ceramic coating (500 μm), a higher U_50_ value of 88 kV is obtained. This is mainly due to that YSZ has a lower permittivity, which can generate a more uniform electric field distribution between the rod and plane electrodes, bringing about a higher breakdown voltage.

## Introduction

In gas insulated switchgear (GIS), sulphur hexafluoride (SF_6_) is the most commonly applied insulation gas because of its unique electrical and thermal properties^1,2^. For instance, it has relatively high electrical breakdown strength due to the strong electronegativity. Moreover, as compared to that of other insulation gases, the arc extinguishing and cooling properties are excellent. Apart from the outstanding electrical and thermal properties, the boiling point of SF_6_ is relatively low in comparison with other electronegative gases. Moreover, SF_6_ is not ozone depleting and toxic^3^. Nevertheless, the Global Warming Potential (GWP) of SF_6_ gas is very high. As compared to that of CO_2_, it is about 23,000 times higher. On this account, the legal regulations of the emission for SF_6_ gas are fairly strict. Now, users of SF_6_ should carefully record the storage amount of SF_6_ gas. During arcing, it can be dissociated into detrimental by-products, which are toxic and can damage spacers. Besides, the costs of production, disposal and handling are relatively high on account of the above mentioned regulations^4,5^. Hence, it is imperative to replace SF_6_ with alternative gas, which should be relatively low cost and environmentally friendly. Meanwhile, it should have sufficiently low boiling point to prevent condensation in working conditions and good dielectric performance to minimize the size increase of GIS designs^6–9^. However, the breakdown strength of most readily available environmentally friendly alternatives, for instance, N_2_, CO_2_ and air, is poor.

There are two methods can be applied to enhance the breakdown strength of GIS. Firstly, developing an alternative gas which is environmentally friendly and has an excellent breakdown performance^10,11^. The theme of this study does not focus on the developing alternative gases. Instead, the focus is on the easily acquired and low cost gas of N_2_. The second way to enhance breakdown voltage, when applying such an eco-friendly but weak insulation gas, is the usage of dielectric coating^12–19^. Although it has been demonstrated that the breakdown strength can be improved through applying dielectric coating on the electrode’s surface, up to now, the available coating materials are very limited. For further increasing the breakdown strength, it is essential to enlarge the range of coating materials.

Generally, ceramic is a very promising coating material because of its outstanding characteristics, such as high chemical and thermal stability^20,21^. Zirconium oxide, as one of the most important ceramic materials, is widely studied including its structure and property and utilized as thermal coating since it has superior mechanical properties, for instance, high strength and fracture toughness combined with good wear resistance and, above all, a thermal expansion coefficient close to that of metallic substrates^22–24^. However, it should be noted that pure ZrO_2_ is not suitable for the ceramic coating as it exhibits different phase at different temperatures, i.e. monoclinic (m-phase below 1170 °C), tetragonal (t-phase between 1170 and 2370 °C) and cubic (c-phase above 2370 °C). The phase transformation from tetragonal to monoclinic is accompanied by a significant volume expansion of approximately 3–5 vol%, which will induce the catastrophic cracking^25,26^. Therefore, in order to successfully apply this material in thermal spraying, it is necessary to solve the problem of how to stabilize the desirable phase in the deposit. In ZrO_2_, the theoretical ratio of the ionic radius of the cation to that of O^2−^ for fully ionic packing is 0.73 at room temperature, but the real ratio is 0.59. Hence, doping other element at Zr site is an efficient way to stabilize the high temperature cubic phase to room temperature by the formation of solid solutions, such as Y element, which has a bigger ionic radius compared to that of Zr^4+^. Generally, for lattice stabilization, the concentration of Y_2_O_3_ should be larger than 8 mol%. Besides, Al_2_O_3_ is always introduced into ZrO_2_ (zirconia-toughened alumina, ZTA) to improve the mechanical property utilizing the recognized toughening mechanisms, and ZTA is also a good candidate for coating material^27–32^.

In 1986, a supersonic plasma spraying system (PlazJet) was invented by Browning Engineering Company^33^. Hybridizing high voltage extended arc plasma with high gas acceleration in an extended nozzle, which led to gas velocity in a supersonic mode at the nozzle exit with high arc power. Nevertheless, during the plasma spraying, the consumption of gas flow and energy in this system was very high, bringing about a high cost for the product. Recently, for the deposition of metallic and ceramic coatings with high performance, national key laboratory for remanufacturing (China) invented an advanced high efficiency supersonic plasma spraying system. In comparison with that of “PlazJet”, energy consumption was significantly lowered^34,35^. Moreover, as compared with that of conventional atmospheric plasma spraying (APS), the in-flight particles can obtain greater energy and momentum in the supersonic atmospheric plasma spraying system since the structural optimization of the internal powder injection and spray gun^36^.

In the present work, with the aim of improving the insulating performance of solid/gas (N_2_) composite insulation system, YSZ ((ZrO_2_)_0.92_(Y_2_O_3_)_0.08_) and ZTA (20 wt% Al_2_O_3_/ZrO_2_) were used as dielectric coating materials at the first time. In order to compactly coat ceramic powders on the target electrode, high efficiency supersonic atmospheric plasma spraying (SAPS) technique was adopted. Moreover, lightning impulse (LI) voltage were applied to investigate the breakdown performance. Additionally, varied characterizations were performed to inspect the YSZ and ZTA ceramic coatings. The authors believe that the results obtained in this study are helpful for the design of the solid/gas composite insulation system.

## Experimental

**Figure 1:**
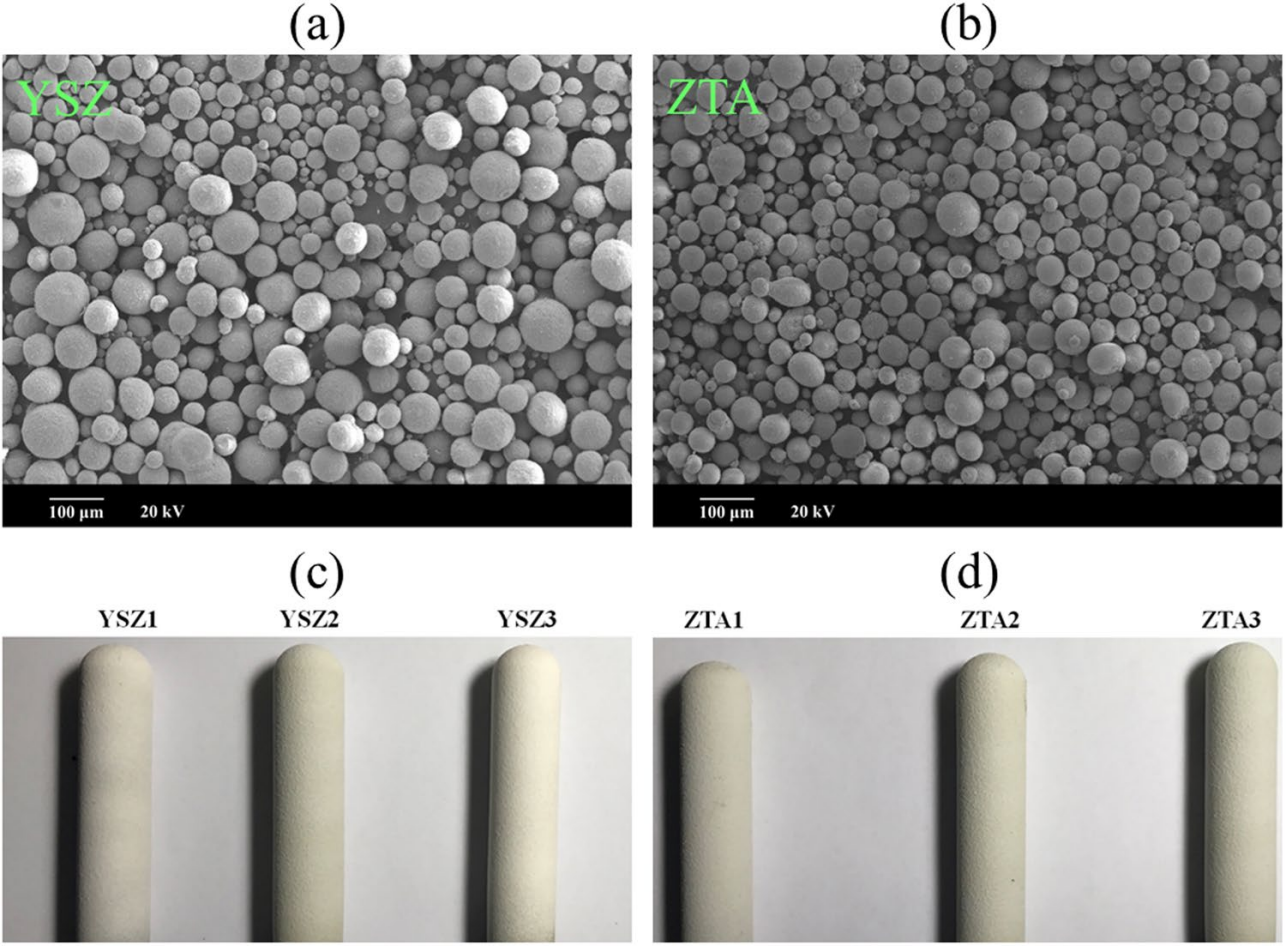
SEM micrographs of the as-received (**a**) YSZ and (**b**) ZTA powders. Images of (**c**) YSZ and (**d**) ZTA coatings (YSZ1 and ZTA1: 300 μm, YSZ2 and ZTA2: 400 μm, YSZ3 and ZTA3: 500 μm).

To increase the adherence capability between coating and substrate, the rod electrode substrate was ultrasonically cleaned and then grit-blasted with alumina powder. The surface roughness (*R*_a_) of the grit-blasted substrate was about 6.3 μm, which was measured by a profilometer (TR 240, Beijing Time Group, Beijing, China). The commercial spherical YSZ and ZTA powders (Haoxi Technical Co., Ltd., China) with particle size 10–100 μm, as shown in Fig. 1a,b, were used as coating materials. The YSZ and ZTA coatings, as shown in Fig. 1c,d, were deposited by high efficiency supersonic atmospheric plasma spraying (SAPS). The advanced SAPS system was equipped with a novel SAPS gun with a Laval nozzle. The length and exit diameter of nozzle were 4.4 and 0.6 cm, respectively. The powders were injected into the plasma jet via an internal injection port with inlet diameter of 0.2 cm, which was inside the nozzle and directed perpendicular to the plasma jet. During the spraying process, the substrate was cooled by compressed air and the temperature monitored by a thermocouple varied between 100 and 200 °C. Besides, the spray parameters are summarized in Table 1.

**Table 1:** Spray parameters for YSZ and ZTA coatings.

Parameters	Current (A)	Voltage (V)	Primary gas, Ar (slpm)	Second gas, H_2_ (slpm)	Powder feed rate (g/min)	Spray distance (mm)
Samples	400	160	60	20	40	100

The measurements of the breakdown voltages were conducted for a rod-plane electrode arrangement. The electrodes of 14 mm and 50 mm in diameters were made of stainless-steel and were centered axially inside a cylindrical polymethyl methacrylate vessel. The vessel was filled with high purity N_2_ (~0.06 Mpa) and gas gap was 2 cm, which could be adjusted by rotating the rod electrode. The standardized lightning impulse (LI) voltage (1,2/50 μs) was supplied by a lightning impulse generator. During the tests, positive polarity impulse voltage was applied on rod electrode based on the up-down method. To evaluate the insulation performance, 50% breakdown voltage (U_50_) of coated electrode and bare electrode was compared.

X-ray diffraction measurements of as-received particles and nanocomposites were performed on a Bruker D2 Phaser diffractometer using a Cu Kα wavelength (1.54 Å) as radiation source, and the 2*θ* ranges of the data were taken from 20° to 80°. X-ray photoelectron spectroscopy (XPS) was carried out with an ESCALAB Xi+ spectrometer, using AI Kα excitation radiation (hγ: 1253.6 eV). The micromorphology of the sample after breakdown tests was acquired by field emission scanning electron microscopy (FESEM, FEI quanta 600, USA). Thermogravimetric analysis (TGA) was inspected using Mettler Toledo TGA/DSC 3 thermo-analyzer in nitrogen atmosphere. The dielectric constant and dielectric loss of the samples were measured via Concept 80 instrument in the temperature range of 0–140 °C under N_2_, and each kind of sample was parallelly measured three times to increase reliability. Before dielectric characterization, Au electrodes were sputtered onto both sides of the specimens via sputtering technique to acquire a good contact between the measuring electrodes and samples.

## Results and Discussion

**Figure 2:**
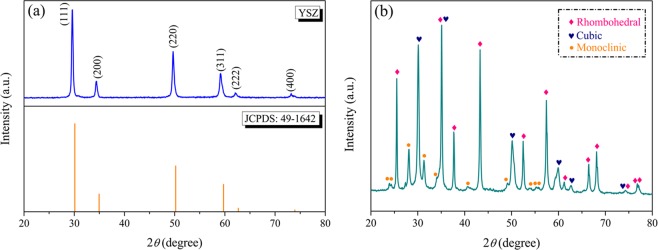
XRD patterns of (**a**) YSZ and (**b**) ZTA powders at room temperature.

XRD technique was employed to investigate the phase of YSZ sample. Figure 2a displays the XRD pattern of as-received YSZ powder at room temperature. By comparison, the XRD pattern matches very well with the standard JCPDS card 49–1642, implying the as-received sample has a cubic crystalline structure, which belongs to space group Fm-3m (225). After a qualitative analysis of the XRD data, the lattice parameter of 5.143 Å was obtained, which is in agreement with previously reported results^37^. Density of 6.069 g cm^−3^ was acquired by using the following formula:

1 $$\rho =\frac{W\times Z}{V\times 0.6022169}$$

where *W* is the formula mass, *Z* is the number of formula units per unit cell and *V* is the volume calculated from unit cell constants. Moreover, the crystallite size is calculated to be 19 nm from Scherrer equation by using (111) peak of the cubic form in the XRD pattern.

2 $$D=\frac{K\times \lambda }{\beta \times \,\cos \,\theta }$$

where *K* is the Scherer constant, *λ* is the average wavelength of Cu Ka radiation, *β* is the full-width-half-maxima (FWHM) of the diffraction peak, and *θ* is the Bragg diffraction angle. XRD pattern of the ZTA powder is shown in Fig. 2b. The presence of rhombohedral phase of Al_2_O_3_ is confirmed by the presence of a high intensity peak at 2*θ* = 35°. Also, the diffraction peaks corresponding to cubic and monoclinic phases of ZrO_2_ can be observed. The average crystallite sizes of ZrO_2_ and Al_2_O_3_ were 21 and 29 nm for ZTA.

**Figure 3:**
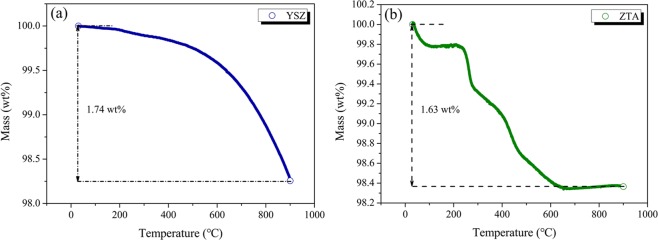
TGA curves of (**a**) YSZ and (**b**) ZTA.

TGA measurement was performed on the as-received YSZ and ZTA powders in the temperature range of 30–900 °C at a heating rate of 5 °C min^−1^ in N_2_, and the obtained TG curves are shown in Fig. 3. With respect to YSZ ceramic, as displayed in Fig. 3a, the initial slight weight loss occurring before 300 °C corresponded to the evaporation of water, while the distinct weight loss appearing between 300 and 900 °C was mainly due to the formation of oxygen vacancies namely the reduction of metal oxides and escape of oxygen ions in the lattice^38^. Within the whole measured temperature, the total weight loss was 1.74%. Regarding the ZTA sample, as shown in Fig. 3b, a major weight loss occured in the temperature range 200–650 °C, as the temperature increased further, the weight got close to stable. At 900 °C, the total weight loss was 1.63%. As a whole, both YSZ and ZTA have good thermal stability and are suitable for applying as thermal spraying materials.

**Figure 4:**
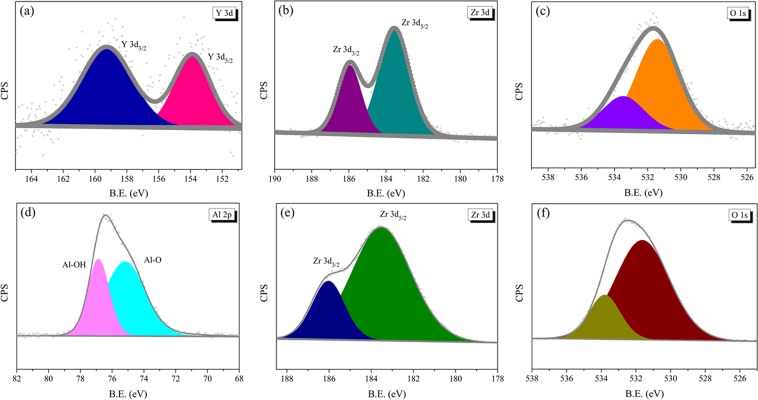
(**a**) XPS spectrum of Y 3d for YSZ. (**b**) XPS spectrum of Zr 3d for YSZ. (**c**) XPS spectrum of O 1s for YSZ. (**d**) XPS spectrum of Al 2p for ZTA. (**e**) XPS spectrum of Zr 3d for ZTA. (f) XPS spectrum of O 1s for ZTA.

To investigate the surface state information of YSZ and ZTA, the samples were analyzed by X-ray photoelectron spectroscopy (XPS). The high-resolution results of Y 3d, Zr 3d and O 1s for YSZ are shown in Fig. 4a–c, while the corresponding results of Al 2p, Zr 3d and O 1s for ZTA are displayed in Fig. 4d–f, respectively. During the fitting, the Lorentzian-Gaussian ratio was fixed and background was subtracted basing on the Shirley method. The binding energies obtained in the XPS analysis were standardized by using C1s as the reference at 284.8 eV.

As can be seen from Fig. 4a, the Y excitation gives rise to two peaks, i.e. Y 3d_5/2_ and Y 3d_3/2_ at a binding energy of 153.9 and 159.3 eV, respectively, similar to those reported by other authors^39,40^. As to Al 2p of ZTA (Fig. 4d), the peak centred at ~75.2 eV can be ascribed to the Al-O bond, while the peak occurring at ~76.9 eV can be attributed to the Al-OH bond, respectively^41^. The binding energies of Zr 3d_5/2_ and Zr 3d_3/2_ for YSZ (Fig. 4b) are 183.6 and 185.9 eV, while the corresponding results of ZTA (Fig. 4e) are 183.5 and 186.1 eV, respectively. The O 1s peak, as indicated in Fig. 4c and f, is relatively broad and asymmetric due to it derives from different types of oxygen species. Further deconvolution reveals two distinct components. The high peak stands for oxygen ions bounded by several metal atoms, while the surface adsorbed oxygen species promote the formation of low peak^42,43^. The binding energies of YSZ and ZTA samples are summarized in Table 2.

**Table 2:** Binding energies of YSZ and ZTA samples.

Sample	Binding energy (eV)					
	Y 3d_3/2_	Y 3d_5/2_	Zr 3d_3/2_	Zr 3d_5/2_	Al 2p	O 1s
YSZ	159.3	153.9	185.9	183.6	—	531.7
ZTA	—	—	186.1	183.5	76.6	532.5

**Figure 5:**
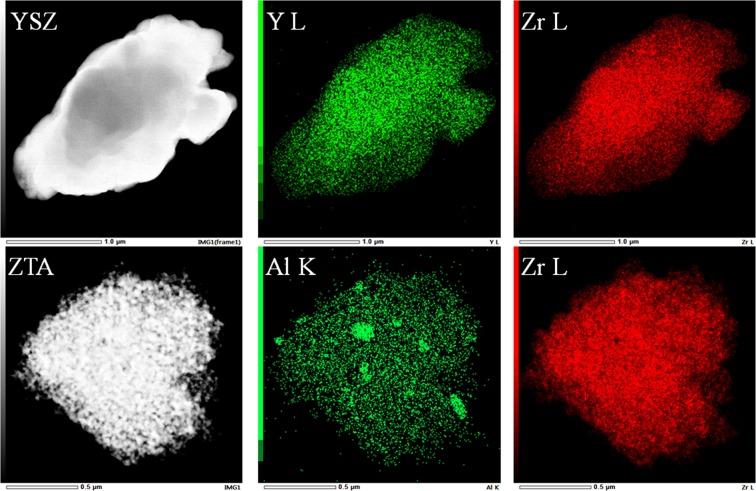
SEM images and the corresponding element mappings of YSZ and ZTA.

**Figure 6:**
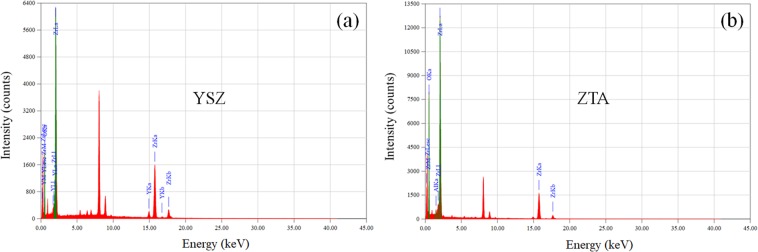
EDS spectra of (**a**) YSZ and (**b**) ZTA.

Homogeneous distribution of the particles is one of the important factors in thermal coating. Figure 5 shows the elemental distribution maps obtained from EDS system for YSZ and ZTA samples. It can be observed that each element is evenly distributed in the measured samples. Furthermore, EDS spectra of the elements in the YSZ and ZTA powders are illustrated in Fig. 6. Apart from the elements of substrate, the EDS profiles demonstrate the composition of Zr, Y and O elements for YSZ, and Zr, Al and O elements for ZTA, which are in good accordance with the XPS results.

In order to understand the dielectric properties of polymer materials, we are usually interested in the complex permittivity ε*, which relates to a material’s ability to respond to the electric field by its polarization. The complex permittivity is associated with the real part *ε*′ and imaginary part *ε*″.

3 $${\varepsilon }^{\ast }=\varepsilon ^{\prime} -j\varepsilon ^{\prime\prime} $$

4 $$\tan \,\delta =\frac{\varepsilon ^{\prime\prime} }{\varepsilon ^{\prime} }$$

The real part *ε*′ represents the charge storage capacity of the materials, while the imaginary part *ε*″ and tan*δ* characteristics signify the electrical conduction and dielectric relaxation behaviors apart from manifesting a measure of the dielectric losses of the material. In the present work, the complex permittivity and tan*δ* were measured in the frequency range from 10^−2^ to 10^6^ Hz under N_2_ atmosphere.

**Figure 7:**
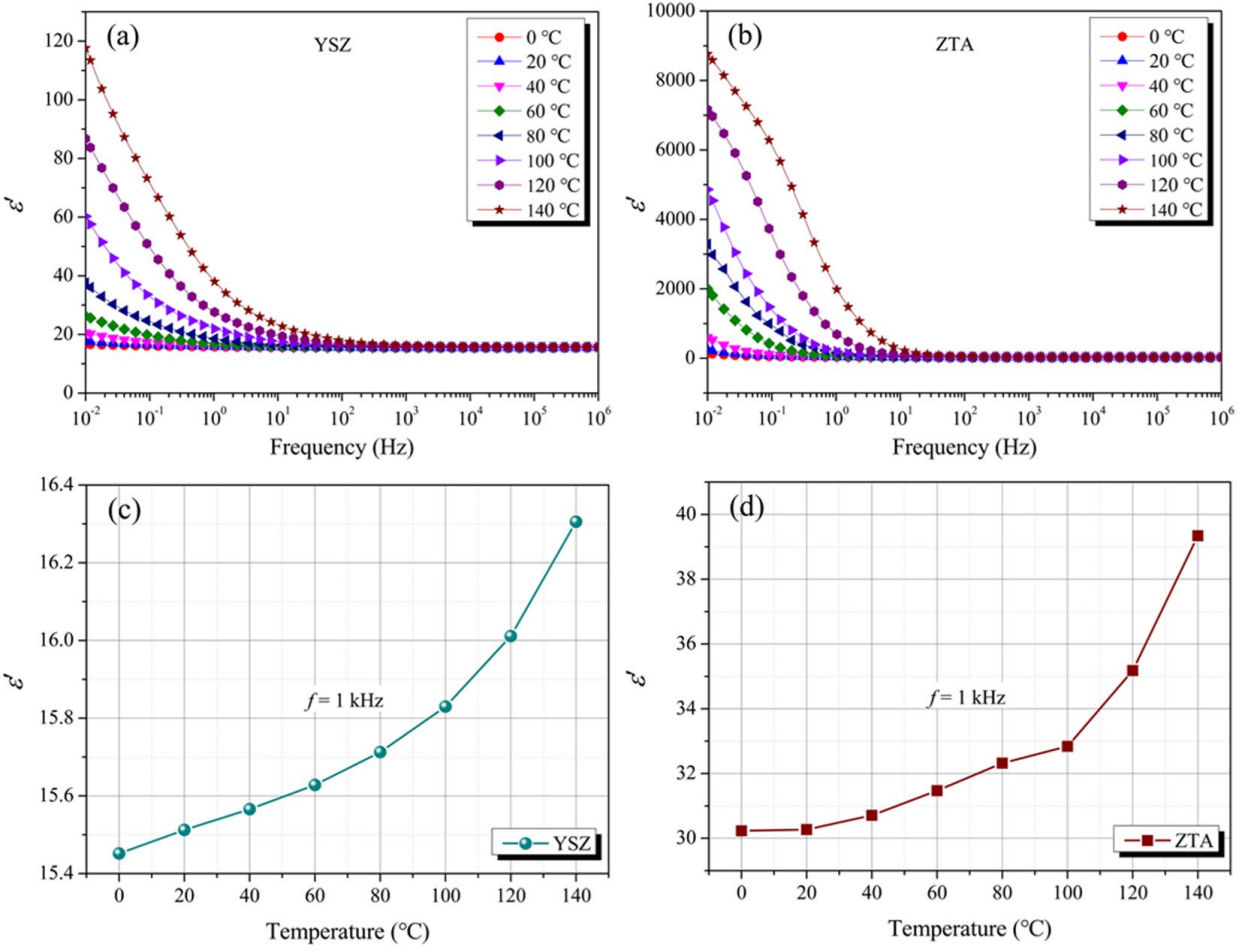
ε′ of (**a**) YSZ and (**b**) ZTA as a function of frequency at tested temperatures. *ε*′ of (**c**) YSZ and (**d**) ZTA as a function of temperature at 1 kHz.

Figure 7a presents the variation in dielectric constant (*ε*′) of YSZ ceramic as a function of frequency at different temperatures. It can be found that the dielectric constant decreases with increasing frequency. This is due to that, at high frequencies, the polarizability (electronic, ionic and orientation polarization) and electric displacement of the dielectric material cannot keep up with the variation of electromagnetic field, which will induce the phenomenon that *ε*′ decreases with the increase of frequency^44^. With regard to ZTA ceramic, as shown in Fig. 7b, it exhibits similar variation tendency of *ε*′ to that of YSZ ceramic in the measured frequency. Moreover, the temperature dependency of *ε*′ for YSZ and ZTA ceramics at 1 kHz are displayed in Fig. 7c and d, respectively. Obviously, *ε*′ increases with the increase in temperature. For YSZ, *ε*′ increases from 15.45 at 0 °C to 16.31 at 140 °C. As compared to that of YSZ, ZTA shows higher *ε*′ values in the temperature range 0–140 °C, and the *ε*′ varies between 30.23 and 39.34.

**Figure 8:**
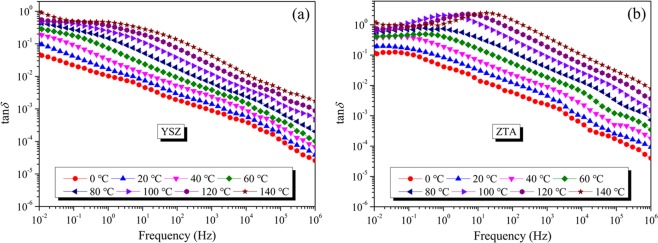
The variation in tan*δ* of (**a**) YSZ and (**b**) ZTA as a function of frequency between 0 and 140 °C.

Dielectric loss tangent (tan*δ*) spectra of YSZ and ZTA samples measured at different temperatures are displayed in Fig. 8. As for YSZ, as presented in Fig. 8a, the dielectric loss tangent decreases with an increase of frequency and no dielectric loss peak is observed under the present experimental conditions. Moreover, it can be observed that the dielectric loss tangent increases as the temperature increases. For a system with conductivity relaxation, the relationships among dielectric loss tangent, conductivity and frequency can be described by the following equation^45^:

5 $$\tan \,\delta =\frac{\sigma }{{\varepsilon }_{0}\varepsilon ^{\prime} 2\pi f}$$

where *σ* is conductivity, *ε*_0_ is vacuum dielectric constant, and *f* is frequency. Since *σ* is strongly dependent on temperature, the dielectric loss tangent is strongly dependent on temperature as well. The relationships between dielectric loss tangent, frequency and temperature in Fig. 8a show the features of conductivity relaxations. It suggests that even at low temperatures conductivity relaxations occur in YSZ ceramic. As can be seen from Fig. 8b, when the temperature reaches to 100 °C, a dielectric loss peak appears at ~1 Hz for ZTA ceramic, which may be ascribed to the existence of electric dipoles or vacancy complexes. Through further observation, it can be found that the dielectric loss peak shifts to a higher frequency as the temperature increases.

**Figure 9:**
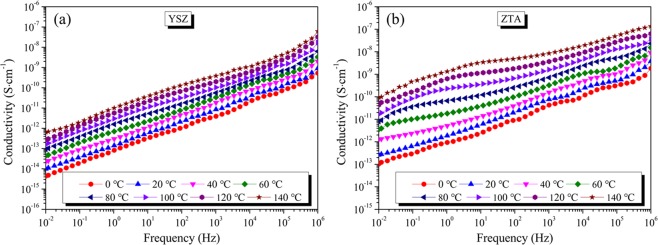
Conductivity of (**a**) YSZ and (**b**) ZTA as a function of frequency at tested temperatures.

In order to further investigate the influence of temperature on dielectric properties of YSZ and ZTA ceramics, alternating current (AC) conductivity measurements were performed in the temperature range from 0 to 140 °C. Figure 9 displays the frequency dependence of AC conductivity (*σ*_ac_) of YSZ and ZTA. The nature of variation of *σ*_ac_ with frequency shows dispersion throughout the measured frequency region, and the conductivity increases with increasing in frequency, which may be associated with space charge in materials. Moreover, as the temperature increases, the enhanced conductivity is probably attributed to the absorbed heat energy, which contributes to the mobility of charge carriers. At 10^−2^ Hz, the conductivity of YSZ is 9.9 × 10^−15^ S·cm^−1^ at 20 °C, suggesting that YSZ is a good insulating material. Regarding the ZTA ceramic, under the same conditions, a conductivity value of 2.6 × 10^−13^ S·cm^−1^ is obtained.

**Figure 10:**
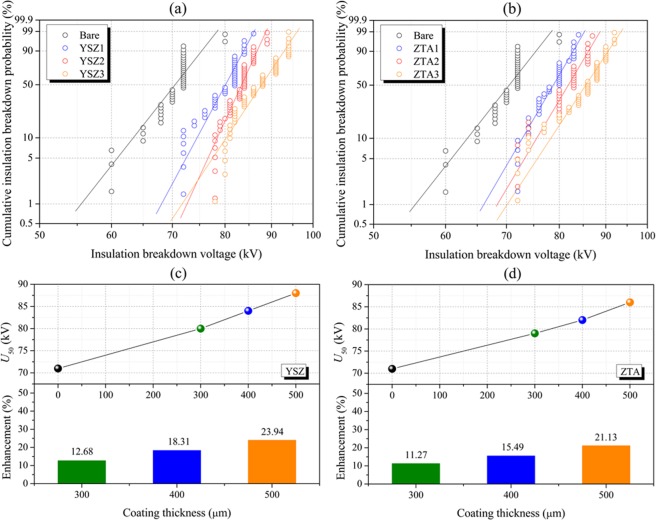
Weibull plots of insulation BDV for tested electrodes using (**a**) YSZ and (**b**) ZTA as coating materials. U_50_ and enhancement of the tested electrodes using (**c**) YSZ and (**d**) ZTA as coating materials.

Figure 10a,b shows the Weibull distribution of breakdown voltages at lightning impulse (LI) for bare electrode and electrodes coated with YSZ and ZTA dielectric materials. During the measurements, the environmentally friendly N_2_ gas with a relative pressure of 0.06 MPa was applied, while the gap distance was set to 2 cm. To determine coating thickness, the measuring field was equidistantly divided into six sections. For each section, 20 points were randomly selected and the corresponding thickness values were recorded. *T*_i_ (*i* = 1, 2, 3, 4, 5, 6) was the average value of the recorded values, and the coating thickness was the mean value of the six *T*_i_ values. The obtained thickness of YSZ1 (ZTA1), YSZ2 (ZTA2) and YSZ3 (ZTA3) is about 300, 400 and 500 μm, respectively.

As displayed in Fig. 10a,b, it can be found that the 50% breakdown voltage (U_50_) of uncoated electrode is remarkably lower than that of YSZ and ZTA coated electrodes, suggesting the breakdown strength can be efficiently enhanced after the application of dielectric coating, and YSZ and ZTA ceramics are potential candidates as dielectric coating material. Furthermore, the results reveal that the U_50_ value increases with the increase in coating thickness. For observation, the thickness dependence of 50% breakdown voltage as well as enhancement for YSZ and ZTA coated electrodes are coplotted in Fig. 10c,d, respectively. For the electrode with YSZ coating material, the U_50_ value increases from 80 kV for the electrode with YSZ1 coating material to 88 kV for the electrode with YSZ3 coating material, which is enhanced approximately 23.94% compared to that of bare electrode. The obtained U_50_ values of ZTA1, ZTA2 and ZTA3 coated electrodes are 79, 82 and 86 kV, respectively. By comparison, it can be demonstrated that YSZ ceramic coating shows a better breakdown performance under the similar testing conditions, that is to say, YSZ ceramic is a prior candidate.

**Figure 11:**
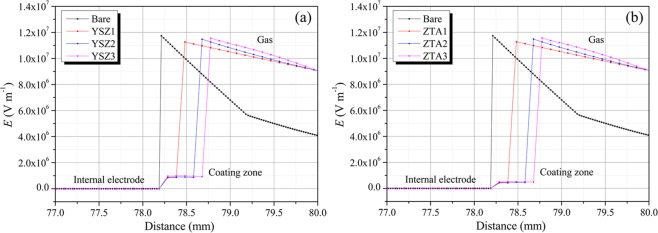
Electric field distribution in a rod-plane configuration with (**a**) YSZ and (**b**) ZTA ceramic coatings. Data before 77 mm are omitted for a clear observation, since *E* value of the internal electrode is 0.

Figure 11a presents the electric field distribution in the coaxial rod-plane electrode system with YSZ ceramic coating of different thickness, which was simulated by commercial software ANSOFT. For comparison, the electric field distribution of bare electrode is also shown in the figure. Regarding bare electrode, as shown in the black line, the highest electric field strength of 1.1738 × 10^7^ V m^−1^ is located at the tip of the rod electrode where the electric field is most divergent. After coating the ceramic coating on the surface of electrode, the electric field distribution changes in compassion with that of bare one. It can be found that the electric field at the electrode surface in the coating is lowered since the ceramic coating has a higher permittivity than that of gas. Moreover, at the coating gas interface the electric field strength exhibits a jump which is also caused by the higher permittivity of the ceramic coating. Even with the sudden increase of the electric field strength at the surface of ceramic coating, the maximum E value in the gas is lowered as compared to that of the bare electrode. In coating zone, there is no significant change in electric field strength as the coating thickness increases, while the E value increases with the increase of coating thickness at the gas coating interface, and E values of 1.1260 × 10^7^,1.1475 × 10^7^ and 1.1559 × 10^7^ V·m^−1^ are obtained for YSZ1, YSZ2 and YSZ3, respectively. Moreover, as the transition from the coating/gas interface to the gas occurs, the electric field strength of ceramic coatings of different thickness tends to be consistent. Similarly, there also are two jumps of electric field strength occurs in ZTA coated electrode as displayed in Fig. 11b. The first jump appears at the interface of electrode/coating, while the second jump occurs at coating/gas interface. As compared to those of YSZ, higher E values of 1.1273 × 10^7^, 1.1484 × 10^7^ and 1.1568 × 10^7^ V·m^−1^ are acquired for ZTA1, ZTA2 and ZTA3 at the interface between coating and gas, respectively. By comparison, under the same thickness, YSZ coating with lower permittivity shows higher E value in coating zone, while it has lower E value in gas. That is to say, the difference of E value between gas and coating is smaller. In this condition, a more uniform electric field will be obtained, resulting in a better breakdown performance of gas-coating insulation system. In consideration of the parallel test environment and similar thickness of ceramic coating, it may be concluded that the breakdown performance of gas-coating insulation system has a close relationship with the dielectric property of the applied coating, and a lower permittivity of dielectric coating will bring about a higher breakdown voltage.

**Figure 12:**
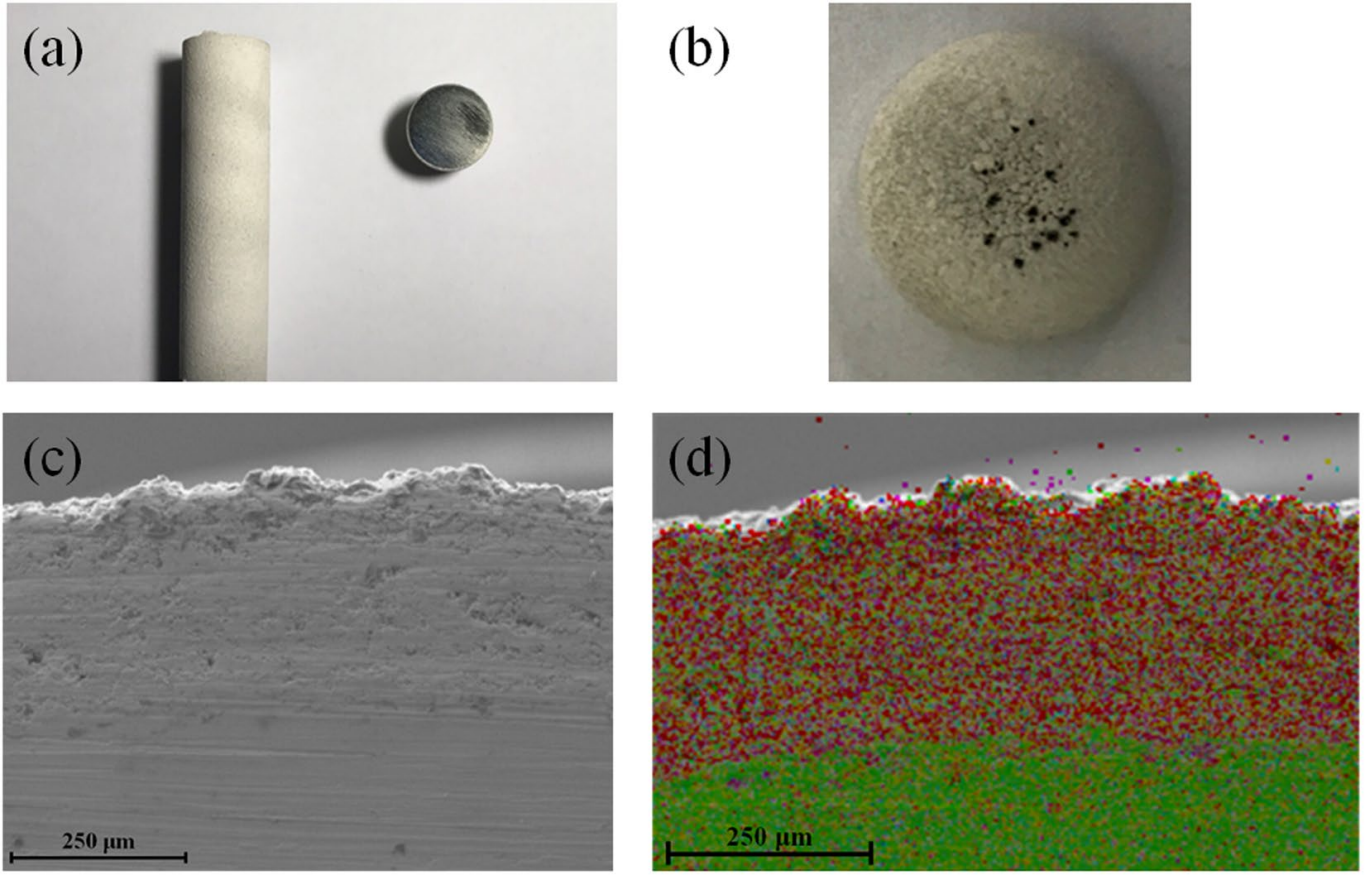
(**a**)The typical image of the coated electrode after cutting. (**b**) Image of the head part of the coated electrode after breakdown tests. (**c**) Cross section view and (**d**) the corresponding EDS result of the electrode with coating.

After the lightning impulse breakdown tests, SEM was performed on the coated electrode. For observation, the target sample was cut from the parent electrode with coating via cutting machine, as shown in Fig. 12a. Then, the electrode head was adhered to the sample stage through conductive adhesive tape. As can be seen from Fig. 12b, some parts of the electrode became gray-black color, meanwhile, some black spots with different size appeared around the top of the coated electrode. SEM result, as displayed in Fig. 12c, demonstrates that ceramic coating thickness is ~300 μm, which is in consistence with the result obtained from coating thickness gauge. For clearly observing the interface between ceramic coating and electrode substrate, the corresponding EDS result is presented in Fig. 12d. It can be observed that the ceramic coating is closely combined with the stainless steel electrode substrate, implying that there was no delamination or detachment appeared during the LI breakdown tests.

**Figure 13:**
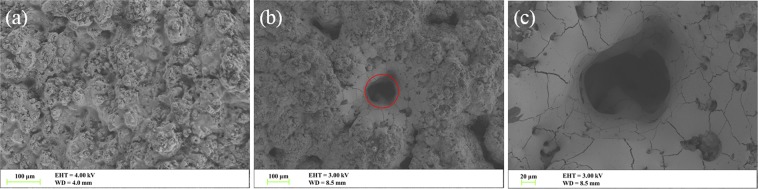
The typical surface views of the electrode with coating (**a**) before and (**b**) after LI BD tests. (**c**) Enlargement view of (**b**).

The typical SEM images of the ceramic coated electrodes before and after the breakdown tests are shown in Fig. 13a,b, respectively. As displayed in Fig. 13b, several distinct breakdown punctures with different shapes and sizes can be clearly seen. The relative small punctures seem to be caused by only a single or a few discharges, while the largest puncture (about 145 μm), as the red circle indicated, should be the starting point of multiple discharges. Moreover, the shape of the puncture becomes rounder with the increase of discharge times. When observing the largest puncture more closely, as presented in Fig. 13c, it can be seen that there exists numerous small punctures in the wall of parent puncture, ascribing to the multiple breakdowns occurring from the same location, resulting in an increased melting of ceramic coating material. Generally, in the process of breakdown test, if some position of the insulation material is punctured, the following BDV will be reduced, since the breakdown point can become the continuously punctured position. In addition, after careful observation, it could be found that there were a lot of cracks appeared on the wall of the puncture, which might be attributed to the thermal stress of the material. It is worth noting that the breakdown performance of the gas-coating composite insulation system can be further improved through optimizing the coating preparation process, enhancing the coating density, improving the coating melting point, *et al*.

Generally, the applied external electric field can induce the movement of charge in ceramic coating material. Since the existence of defects in ceramic material, such as porosity, crack and grain boundary, the charge tends to accumulate at defects under external electric field, resulting in the rapid rise of local voltage. When the threshold value is attained, the local breakdown will happen. At the same time, the electric energy in the breakdown process can be converted into heat energy, which can lead to the sudden increase of temperature in the local part. On this condition, the initial structure of ceramic coating can be damaged. Moreover, the formed crack will expand along the direction of the electric field, and this crack extension provides a path for the movement of charge, while the charge movement provides energy for the further crack extension. Finally, the crack is connected with each other, bringing about the final complete breakdown.

## Conclusions

In the present study, high efficiency supersonic atmospheric plasma spraying (SAPS) method was applied to coat YSZ and ZTA ceramic materials and the lightning impulse breakdown behaviour of YSZ and ZTA dielectric coatings was investigated in rod-plane setup under environmentally friendly N_2_ atmosphere. Moreover, a series of characterization were carried out to inspect the YSZ and ZTA ceramics. The obtained results indicate that both YSZ and ZTA ceramics have good thermal stability. In the measured frequency range of 10^−2^–10^6^ Hz, the dielectric constant (*ε*′) decreases with the increase of frequency. At 1 kHz, *ε*′ increased from 15.45 to 16.31 for YSZ at measured temperatures, while ZTA had higher *ε*′ values in the temperature range 0–140 °C, and it varied between 30.23 and 39.34. As compared to that of bare electrode, the 50% BDV (U_50_) was efficiently enhanced after coating YSZ and ZTA ceramic coatings, suggesting YSZ and ZTA are potential candidates as dielectric coating materials to improve the insulation performance of solid/gas composite insulation system. Moreover, the U_50_ increased with the increase of ceramic coating thickness. For the electrode with ZTA3 (500 μm), the U_50_ was 86 kV. For YSZ3 ceramic coating, a higher U_50_ value of 88 kV was acquired. This is mainly due to that a more uniform electric field distribution will be acquired when the permittivity is lower, leading to a higher breakdown voltage. That is to say, YSZ ceramic is a better dielectric coating material.

## Data Availability

The authors declare that the data is available, and can be provided at any stage.
